# Induced abortions among Chinese adolescent girls

**DOI:** 10.1186/s12905-023-02754-w

**Published:** 2023-11-13

**Authors:** Tian Wang, Lei Si, Quanbao Jiang

**Affiliations:** 1 School of Public Policy and Administration, Institute for Population and Development Studies, Xi’an Jiaotong University, Xi’an, China; 2 School of Health Sciences, Western Sydney University, Campbelltown, NSW Australia

**Keywords:** Induced abortion, Adolescent girls, Unmarried women, China

## Abstract

**Background:**

Induced abortion among adolescent girls is a global public health issue and a serious challenge in China, but still remains under-examined. We aimed to examine the overall trend and characteristics of induced abortions among Chinese adolescent girls and to investigate the factors associated with induced abortion.

**Study design:**

Based on the 2017 China Fertility Survey, this study examined the trend and characteristics of induced abortions among adolescent girls with statistical analysis and multiple indicators of descriptive statistics from period and cohort perspectives, including induced abortion proportion and rate, age-specific cumulative proportion, and age-specific cumulative number of induced abortions in adolescent girls.

**Results:**

Between 1996 and 2016, the proportion and rate of adolescent induced abortions first increased and then decreased, and the mean age at the time of induced abortions among adolescent girls declined. The cumulative proportion of women who had experienced induced abortion at the age of 15–19 in a cohort is less than 2.5% but shows an upward trend. Over 70% of all adolescent induced abortions are premarital. The proportion of women with unintended pregnancy experiences increased and is higher among rural, less educated, and ethnic minority women. Similarly, the prevalence of induced abortion is higher among adolescent girls who live in rural areas, are less educated, and come from ethnic minorities. The cumulative number of induced abortions and premarital abortions increased with later cohorts.

**Conclusions:**

This study shows an upward trend in adolescent-induced abortion and a decline in the age at the time of the induced abortion. Women in later birth cohort have a higher proportion of having experienced adolescent induced abortion. Adolescent girls who live in rural areas, who are less educated, or who are from ethnic minorities, are more likely to undergo induced abortions at the age of 15–19. More appropriate educational efforts and interventions are urgently needed to reduce the incidence of adolescent induced abortions.

**Supplementary Information:**

The online version contains supplementary material available at 10.1186/s12905-023-02754-w.

## Introduction

Induced abortion among adolescent girls has become increasingly prominent as a global public health issue. Compared with adult women, adolescent girls are experiencing higher rates of unintended pregnancy and facing greater challenges in accessing safe contraception and abortion services [1, 2]. Pregnant adolescents are more likely to seek abortion services from unregulated commercial providers due to the fear of stigma and disclosure, inconvenience, and lack of health insurance associated with public hospitals [3, 4]. With induced abortion experience, adolescent girls are more likely to experience complications compared to adult women [1, 3, 5], and suffer from psychosocial harm, including depression, anxiety, and low self-esteem [5]. Fifteen percent of unsafe abortions worldwide occur among adolescent females under the age of 20, accounting for a substantial proportion of abortion-related deaths [5].

Previous studies on adolescent girls with induced abortion experience have focused on characteristics of adolescent pregnancies, identifying factors such as disadvantaged socioeconomic status and less promising career prospects [6]. Young women with unhappy upbringings, poor material circumstances, disinterest in schooling, and low future expectations and aspirations are likely to become sexually active at an early age and be at risk of getting pregnant [7]. Studies have also shown that young women with low educational attainment are more likely to experience unintended pregnancy and induced abortion [8]. Furthermore, the notion that active schooling engagement, to a certain extent, lessens the risk of unintended pregnancy is supported by studies showing that adolescent dropouts were more likely to get pregnant. Moreover, pregnancy was reported to less likely occur among students during school time than during school holidays [8, 9]. Most pregnancies and induced abortions among adolescents unintendedly occur before marriage. According to the World Health Organization statistics in 2016, there were 21 million pregnancies among adolescent girls aged 15–19 in developing countries, and about half of them were unintended [10]. Unintended pregnancies among younger women usually result in induced abortions because these young women are unmarried and cannot afford to raise a child [11].

In China, unintended pregnancy among adolescents has also become more prevalent—over 90% of which have ended up in induced abortions [12–15]. Although premarital sex has become increasingly acceptable in China, adolescent pregnancy still carries a social stigma in current Chinese society. The annual estimate of adolescent-induced abortions is 6 million, and still increasing [16]. Moreover, the incidence of adolescent repeat induced abortions is also increasing. According to the survey conducted in 297 hospitals in 30 provinces on 2370 women aged 13–19 who underwent induced abortions, 39.11% were repeat induced abortions, and 8.69% were third or higher-order abortions [13].

Despite the importance and effect of adolescent induced abortions, there is a lack of nationally representative descriptions of the overall trend and characteristics of adolescent induced abortions in China due to data unavailability. Drawing on the previous study about induced abortions among 15–49-year-old women [17], we focus on induced abortions among 15–19-year-old adolescent girls, aiming to complement the research on adolescent induced abortions in China. From period and cohort perspectives, the current study examined the trend and characteristics of induced abortions among adolescent girls with multiple indicators and statistical analysis.

## Methods

### Data

The data used in this study were collected from the 2017 China Fertility Survey conducted by the former National Health and Family Planning Commission. Through stratified three-stage probability proportional to size (PPS) sampling, the data were collected by both face-to-face interviews and online surveys [17]. The final valid sample size in this study was 243,951.

We used information on pregnancy history in this survey, including the month and year of the respondent’s first marriage and the end date of each pregnancy, whether each pregnancy was planned or not, as well as the outcome, which was categorized as live birth, stillbirth, spontaneous abortion, or induced abortion (both medical abortion and induced labor). In addition, the survey included sociodemographic characteristics, including age, residence^1^ (urban or rural areas), ethnicity (Han or minority), and education level (junior middle school or below, high school, and college or above). From the data, we excluded women who were over 60 years old at the time of the survey (*n* = 2380, 0.98%), whose first marriage/cohabitation information was missing (*n* = 23, 0.01%), and whose age at first pregnancy was under 15 (*n* = 248, 0.24%). The final sample size in this study is 241,300, including a total of 2343 induced abortions among individuals aged 15–19.

The vast majority of adolescent pregnancies occur among the 15–19 years age group, and only a meager proportion happens among the 10–14 years age group [18]. In addition, the minimum legal age of marriage for Chinese women is 20 years, so we mainly focus on the level of induced abortions among women aged 15–19 years in this paper. Pregnancies were dichotomized as premarital and post-marital by calculating the interval between the end date of each pregnancy and the date of the first marriage [17]. Firstly, we assessed the difference between women in different characteristics for making comparison. Then, we used multiple indicators from period and cohort perspectives based on the previous research [17].

## Statistical analysis

Firstly, we used the chi-square test to assess differences in whether have experienced induced abortion at the age of 15–19 between birth cohorts (15–19, 20–24, 25–29, 30–34, 35–39, 40–44, 45–49, 50–54, and 55–60, according to their age at the time of the survey), residence (urban or rural), education level (junior middle school or below, high school, and college or above), ethnicity (Han or minority group) and marital status (unmarried or married at the time of the survey).

Then we used logit model to test the linear trend in proportion of women who have experienced adolescent induced abortion across different birth cohorts. Specifically, the dependent variable in the model is binary, indicating whether a woman had experienced induced abortion at the age of 15–19 (0 = No, 1 = Yes). The independent variable is birth cohorts (15–19, 20–24, 25–29, 30–34, 35–39, 40–44, 45–49, 50–54, and 55–60 at the time of the survey). The 25–29 birth cohort was used as the reference in each model. Tests were also conducted separately by characteristics (including residence, education level and ethnicity as described above) to examine trends in the proportion of women who have experienced adolescent induced abortion across birth cohorts among women with different characteristics. All statistical analyses were performed using the Stata version 15.

## Period indicators

*Adolescent induced abortion proportion* refers to the proportion of induced abortions in all pregnancies among 15–19 year-old females in 1 year. Pregnancies include the following results: live births, stillbirths, spontaneous abortion, and induced abortion (including drug abortion and induced labor).

*Adolescent induced abortion rate* denotes the number of abortions per 1000 women aged 15–19 years in 1 year in this study.

## Cohort indicators

According to their age at the time of the survey, respondents were grouped into the following 10 cohorts: 18, 19, 20–24, 25–29, 30–34, 35–39, 40–44, 45–49, 50–54, and 55–60 years. Due to the limited space, when making comparisons with multiple indicators, we used the 25–29 years cohort (i.e., women aged 25–29 years at the time of the survey) to represent the later birth cohort. The 45–49 years cohort (i.e., women aged 45–49 years at the time of the survey) represents women in the earlier birth cohort. Women at the age of 25–29 include those who have completed the college or university education, which is one of the factors we focus on in this study, and women aged 45–49 are at the end of childbearing age. There is a twenty-year gap between these two cohorts, which can represent the later birth cohort and earlier birth cohort.

The cohort indicators of this study are as follows:

*Age-specific cumulative proportion of adolescent induced abortion for a cohort* refers to the proportion of women with adolescent induced abortion experiences to all women in a cohort by a specific age before 20. It can be calculated by induced abortion order, which is the proportion of women who have experienced abortion at least “n” times by the corresponding age. In addition, it can be calculated by residence, education level, and ethnicity.

*Age-specific cumulative number of adolescent induced abortions for a cohort* reflects the average number of adolescent induced abortions experienced by women in a cohort by a specific age before 20. It is calculated by dividing the number of adolescent induced abortions experienced by women in a cohort by the total number of women in the cohort. This can also be calculated respectively based on different factors.

## Results

### Statistical analysis

Descriptive statistics and tests for differences between women with different characteristics are shown in Table 1. Overall, there are 0.89% of women in this sample had experienced at least one induced abortion at the age of 15–19. The *P* values show that there are significant differences in cohort, residence, educational level, ethnic groups and marital status at the time of the survey among women have experienced induced abortions at the age of 15–19. Women in later birth cohort, living in rural areas, with lower educational level, from minorities group or married at the time of the survey have a higher proportion of having experienced adolescent induced abortions.

**Table 1:** Descriptive characteristics and chi-square test results of women who have experienced adolescent induced abortions, No.(%)

Characteristics	Total	Whether experienced induced abortions at the age of 15-19		
		No	Yes	*P*-value for chi2 test
Total	241,300	239,163 (99.11)	2137 (0.89)	–
Cohort				
15-19	12,062 (5.00)	12,019 (99.64)	43 (0.36)	< 0.001
20-24	13,765 (5.70)	13,481 (97.94)	284 (2.06)	
25-29	23,936 (9.92)	23,539 (98.34)	397 (1.66)	
30-34	25,572 (10.60)	25,237 (98.69)	335 (1.31)	
35-39	26,066 (10.80)	25,821 (99.06)	245 (0.94)	
40-44	32,497 (13.47)	32,222 (99.15)	275 (0.85)	
45-49	41,986 (17.40)	41,705 (99.33)	281 (0.67)	
50-54	42,075 (17.44)	41,854 (99.47)	221 (0.53)	
55-60	23,341 (9.67)	23,285 (99.76)	56 (0.24)	
Residence				
Urban	92,025 (38.14)	91,373 (99.29)	652 (0.71)	< 0.001
Rural	149,275 (61.86)	147,790 (99.01)	1485 (0.99)	
Education level				
Junior middle school or below	163,445 (67.74)	161,680 (98.92)	1765 (1.08)	< 0.001
High school	40,925 (16.96)	40,627 (99.27)	298 (0.73)	
College or above	36,930 (15.30)	36,856 (99.80)	74 (0.20)	
Ethnic groups				
Han	216,502 (89.72)	214,662 (99.15)	1840 (0.85)	< 0.001
Minorities	24,798 (10.28)	24,501 (98.80)	297 (1.20)	
Marital status				
Unmarried	28,715 (11.90)	28,604 (99.61)	111 (0.39)	< 0.001
Married	212,585 (88.10)	210,559 (99.05)	2026 (0.95)	

*P* values were computed separately for each characteristic and indicate statistically significant differences in different characteristics if *P* < 0.05

The Table 2 shows the odds ratio of having experienced adolescent induced abortions among women in different birth cohorts. The lower odds ratio of women having experienced adolescent induced abortions in the 15–19 birth cohort was because of the right censoring. A substantial number of induced abortions did not occur among women in this birth cohort. Except for women with college education or above, there are statistically significant trend with birth cohorts among women in different characteristics. Women in later birth cohort have a higher odds ratio of having experienced adolescent induced abortions.

**Table 2:** Odds ratio of having experienced adolescent induced abortions among women in different birth cohorts

Birth cohorts	Total	By residence		By education level			By ethnic groups	
		Urban	Rural	Junior middle school or below	High school	College or above	Han	Minorities
	OR (95% CI)	OR (95% CI)	OR (95% CI)	OR (95% CI)	OR (95% CI)	OR (95% CI)	OR (95% CI)	OR (95% CI)
15-19	0.21 (0.15, 0.29)	0.12 (0.06, 0.24)	0.27 (0.19, 0.38)	0.29 (0.20, 0.41)	0.05 (0.02, 0.12)	0.75 (0.17, 3.29)	0.17 (0.12, 0.25)	0.34 (0.19, 0.60)
20-24	1.25 (1.07, 1.46)	1.06 (0.80, 1.41)	1.36 (1.13, 1.63)	1.62 (1.35, 1.95)	1.14 (0.83, 1.55)	1.21 (0.56, 2.61)	1.18 (1.00, 1.41)	1.43 (1.00, 2.03)
25-29	Reference	Reference	Reference	Reference	Reference	Reference	Reference	Reference
30-34	0.79 (0.68, 0.91)	0.79 (0.61, 1.02)	0.81 (0.68, 0.97)	0.72 (0.61, 0.85)	0.59 (0.41, 0.85)	1.73 (0.89, 337)	0.80 (0.68, 0.94)	0.70 (0.48, 1.01)
35-39	0.56 (0.48, 0.66)	0.53 (0.40, 0.71)	0.59 (0.49, 0.72)	0.48 (0.40, 0.57)	0.31 (0.20, 0.49)	1.33 (0.62, 2.82)	0.58 (0.49, 0.69)	0.47 (0.31, 0.71)
40-44	0.51 (0.43, 0.59)	0.53 (0.40, 0.70)	0.49 (0.41, 0.59)	0.37 (0.32, 0.44)	0.35 (0.23, 0.54)	0.72 (0.26, 2.01)	0.53 (0.45, 0.63)	0.37 (0.24, 0.56)
45-49	0.40 (0.34, 0.47)	0.49 (0.37, 0.65)	0.35 (0.29, 0.42)	0.28 (0.24, 0.34)	0.18 (0.11, 0.32)	0.78 (0.26, 2.38)	0.45 (0.38, 0.52)	0.13 (0.07, 0.24)
50-54	0.31 (0.27, 0.37)	0.44 (0.33, 0.59)	0.26 (0.21, 0.32)	0.22 (0.18, 0.26)	0.15 (0.09, 0.26)	0.30 (0.04, 2.29)	0.35 (0.29, 0.41)	0.12 (0.06, 0.23)
55-60	0.14 (0.11, 0.19)	0.19 (0.11, 0.31)	0.12 (0.09, 0.17)	0.11 (0.08, 0.15)	0.05 (0.02, 0.13)	0	0.14 (0.10, 0.19)	0.19 (0.09, 0.39)
p for trend	< 0.001	< 0.001	< 0.001	< 0.001	< 0.001	0.15	< 0.001	< 0.001

The birth cohorts were grouped by the age (numerical variable) of women on the survey date. P values for trend were computed separately by each characteristic and indicate there are statistically significant trend in birth cohorts if *P* < 0.05

### Overall trend of induced abortions at the age of 15–19

**Fig. 1:**
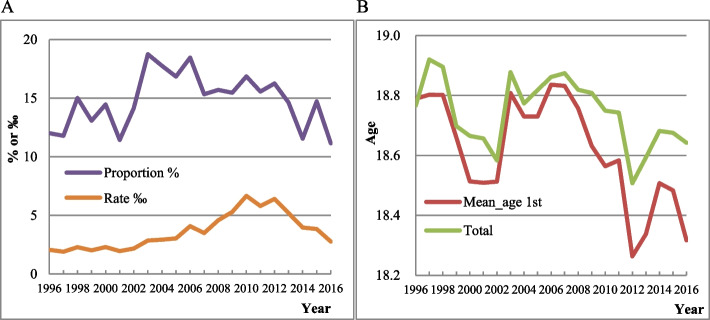
Overall trend of induced abortions among women aged 15-19. (1A) Adolescent induced abortion proportions and rates in 1996-2016. (1B) Mean age of adolescent induced abortions in 1996-2016. Note: Mean_age 1st denotes the mean age at induced abortions among first pregnancies. Total denotes that the mean age at all induced abortions

Figure 1 presents the overall trend of adolescent induced abortion proportion and rate, as well as the mean age at the time of induced abortions among adolescent girls in China. Panel 1A shows that the proportion of adolescent induced abortion fluctuated between 10 and 20% from 1996 to 2016, and there was an overall downward trend after 2006, from 18.46% in 2006 to 11.15% in 2016. Adolescent induced abortion rate shows an upward trend and then declines, with a rate of 2.76‰ in 2016. The numbers in Fig. 1 can be referred to in Supplemental Table 1.

The mean age of adolescent induced abortion has been declining since 2006. As illustrated in Panel 1B, the mean age is mainly concentrated between 18 and 19 years for all induced abortions among women aged 15–19, and it showed a downward trend. Moreover, the mean age at induced abortions among first pregnancies showed a marked decline since 2006.

### Adolescent unintended pregnancy and induced abortion

**Fig. 2:**
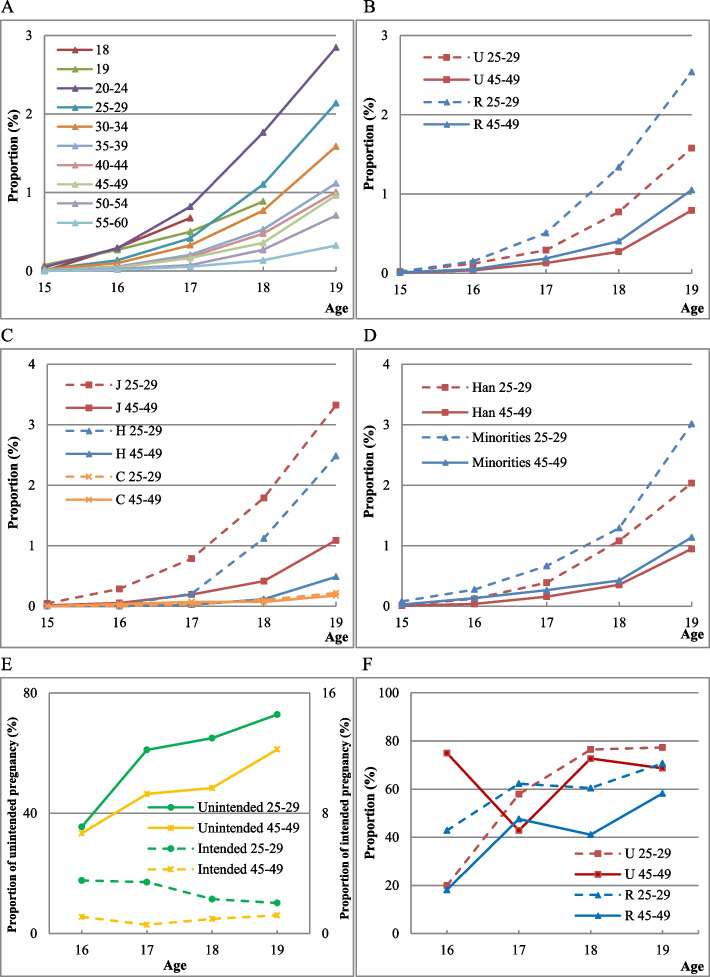
Unintended pregnancies and induced abortion proportions at the age of 15-19. (2A) Cumulative proportion of unintended pregnancies by cohort. (2B) Cumulative proportion of unintended pregnancies by residence. (2C) Cumulative proportion of unintended pregnancies by education. (2D) Cumulative proportion of unintended pregnancies by ethnic group. (2E) Induced abortion proportion of unintended and intended pregnancies. (2F) Induced abortion proportion of unintended pregnancies by residence. Note: U and R represent Urban and Rural respectively; J, H, and C respectively represent junior middle school and below, high school education, college and above. Minority includes all other ethnic groups excluding Han Chinese

Figure 2 presents the trend and characteristics of unintended pregnancies at the age of 15–19 among women in all birth cohorts.

The prevalence of unintended pregnancies among adolescent girls is rising. Panel 2A shows the trend of unintended pregnancies among adolescent girls in different birth cohorts. There is an increasing trend in the proportion of women who experienced unintended pregnancies in adolescence; the later the birth cohort, the higher the proportion of unintended pregnancies. At the time of the survey, 2.85% of women aged 20–24 and only 0.33% of women aged 55–60 had experienced unintended pregnancies by the age of 19. Meanwhile, the unintended pregnancy proportion among women at 18 years old is higher than that among women at 19 years.

Women in rural areas show a higher proportion than women in urban areas in all birth cohorts, as shown in Panel 2B, and the differences between urban and rural areas increased in the late birth cohort.

Women with lower education are more likely to experience unintended pregnancies. Panel 2C shows the differences in adolescent unintended pregnancy among women with different levels of educational attainment. In each cohort, those with lower education have higher unintended pregnancy proportions. For women with the same education, the proportion is lower in the late birth cohort than that in the early birth cohort. This trend is marked especially in women with a high school education or below.

Panel 2D presents the difference in adolescent unintended pregnancies among women from different ethnic groups. The proportion of adolescent unintended pregnancies is higher in the late birth cohort than that in the early birth cohort among both Han and ethnic minority women. In each cohort, ethnic minority women have higher proportions than Han women. Especially in the late birth cohort, the proportion is markedly higher for ethnic minority women than Han women, and the difference increased.

Most adolescent girls will choose induced abortion to terminate unintended pregnancies. As shown in Panel 2E, the induced abortion proportion for unintended pregnancies is much higher than intended, and the proportion is higher in the later birth cohort than that in the early birth cohort. There are two reasons for the high abortion proportion at the age of 15. The first is the high likelihood that pregnancies ended in induced abortion at the age of 15, and the other is the small number of observations (5 unintended pregnancies at age 15 for the 25–29 years cohort, 4 for the 45–49 years cohort in this survey data).

Panel 2F shows the induced abortion proportion of unintended pregnancies between women in urban and rural areas. Among women who experienced unintended pregnancies in adolescence, the induced abortion proportion is higher for urban women than that for rural women, which indicates that women in urban areas are more likely to seek abortion when pregnant at the age of 15–19 years.

### Cumulative proportion of adolescent induced abortions by cohort

#### Age-specific cumulative proportion of first-induced abortion in a cohort

**Fig. 3:**
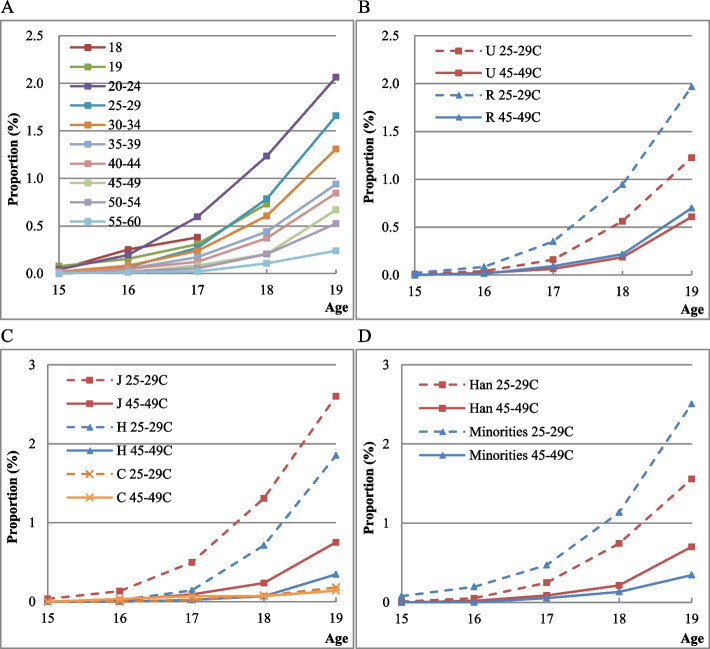
Age-specific cumulative proportion of first induced abortion at the age of 15-19 in a cohort. (3A) By cohort. (3B) By residence. (3C) By education. (3D) By ethnic group. Note: U and R represent Urban and Rural respectively; J, H, and C respectively represent junior middle school and below, high school education, college and above. Minority includes all other ethnic groups excluding Han Chinese

Figure 3 presents the cumulative proportion of women who had experienced their first induced abortion by a specific age in different cohorts.

The prevalence of adolescent induced abortion is increasing in the late birth cohort. Panel 3A shows the cumulative proportion of women who experienced the first induced abortion. In general, the proportion in all birth cohorts is not particularly high but shows an upward trend. The later the birth cohort, the higher the proportion. At the time of the survey, 2.04% of women aged 20–24 had experienced induced abortions by the age of 19, while it was only 0.24% for women aged 55–60. The age-specific cumulative proportion of first induced abortion among women by the age of 18 is also higher than that by 19 years.

The prevalence of adolescent induced abortion is much higher in rural areas. Panel 3B shows the urban-rural differences in the cumulative proportion of women who had experienced the first induced abortion at the age of 15–19. The proportion is higher for women in rural areas than that in urban areas in both the 45–49 and 25–29 years cohort. The disparities between urban and rural areas become larger in the late birth cohort.

The lower the education level, the higher the cumulative proportion of first-induced abortion, as Panel 3C shows. For women with the same level of education, those in the late birth cohort have a higher proportion than those in the early birth cohort. Among women in the late birth cohort, the proportion of women with a high school education or below increased markedly; indicating that the prevalence of induced abortion is rising in low-educated adolescent girls.

The prevalence of adolescent induced abortion varies across ethnic groups in China. As shown in Panel 3D, the proportion is higher among Han women than ethnic minorities in the early birth cohort; while it is higher among ethnic minorities than Han women in the late birth cohort. The proportion is higher in the late birth cohort than that in the early birth cohort among both Han and ethnic minority women, again indicating that the prevalence of adolescent induced abortion is rising in later birth cohorts.

#### Age-specific cumulative proportion of repeat induced abortions by cohort

**Fig. 4:**
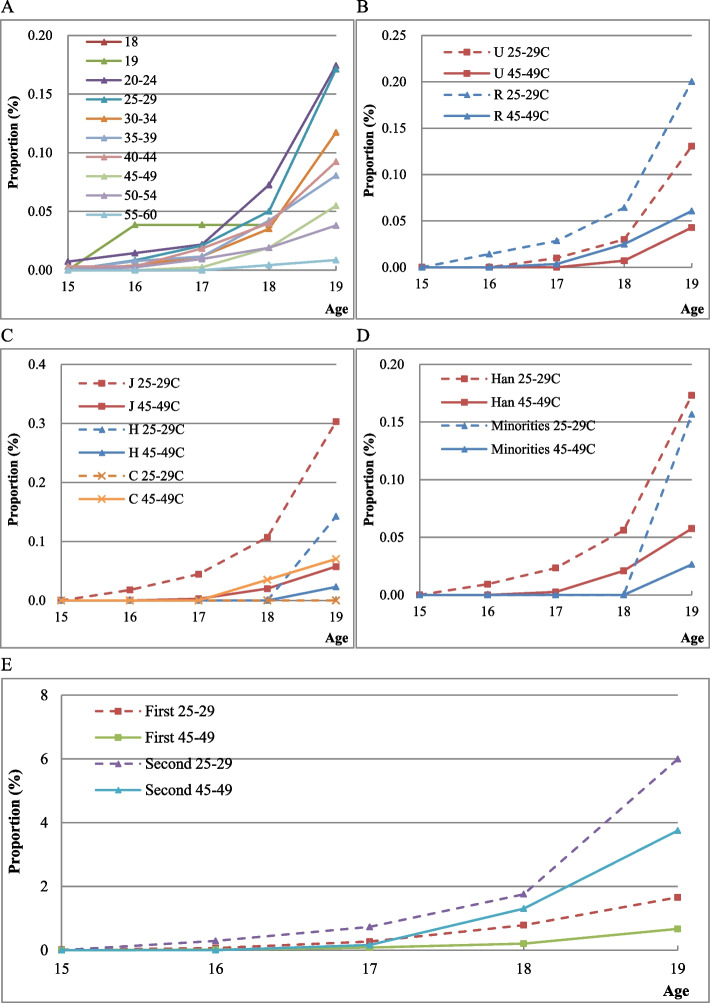
Age-specific cumulative proportion of repeat induced abortions in a cohort. (4A) By cohort. (4B) By residence. (4C) By education. (4D) By ethnic group. (4E) Age-specific cumulative proportion of first induced abortion and progression ratio of repeat induced abortions. Note: In Panel 4E, “First 25-29” refers to women in 25-29 cohort who have experienced the first induced abortion, “Second 25-29” refers to women who have experienced repeat induced abortions among women who had experienced the first abortion in 25-29 cohort

Figure 4 presents the cumulative proportion of women who have experienced repeat (second or more) induced abortions by a specific age.

There is an increasing trend of repeat adolescent induced abortions in the late birth cohorts. As Panel 4A shows, except for the birth cohorts of 18, 19, and 25–29, the later the birth cohort, the higher the repeat induced abortion proportion. Moreover, the proportion of women from the 25–29 years cohort who experienced repeat induced abortions by the age of 19 is higher than that of any other birth cohort.

The urban-rural differences in the cumulative proportion of women with repeat induced abortion experiences at the age of 15–19 are shown in Panel 4B. The proportion is higher in the late birth cohort than that in the early birth cohort in both urban and rural areas. The proportion in rural areas is higher than that in urban areas, which indicates the high prevalence of repeat induced abortions among rural adolescent girls.

The prevalence of repeat induced abortions varies among women with different educational attainments. As shown in Panel 4C, for women with a high school education or below, the proportion is higher in the late birth cohort than that in the early birth cohort. However, for women with a college education or above, the proportion is lower in the late birth cohort than that in the early birth cohort, which indicates that adolescent repeat induced abortions are less likely among women with a higher education.

Han women have higher repeat induced abortion prevalence than ethnic minority women. Panel 4D shows the cumulative proportion of repeat induced abortions among women of different ethnic groups. The proportion is higher in the late birth cohort than that in the early birth cohort among both Han and ethnic minority women. The proportions are higher among Han women than that among ethnic minority women in both early and late birth cohorts.

Panel 4E shows the cumulative proportion for women in different cohorts who experienced the first induced abortion at the age of 15–19. It also shows the progression ratio for women who experienced a second abortion among women who had experienced the first abortion. By the age of 19, more than 3% of women who have experienced their first induced abortion underwent repeat abortions in all birth cohorts. The proportion of repeat induced abortion is 3.77% in the early birth cohort by the age of 19, while it is 6.02% in the late birth cohort, indicating that the prevalence of repeat induced abortion among adolescent girls is increasing.

### Cumulative number of adolescent induced abortions by cohort

**Fig. 5:**
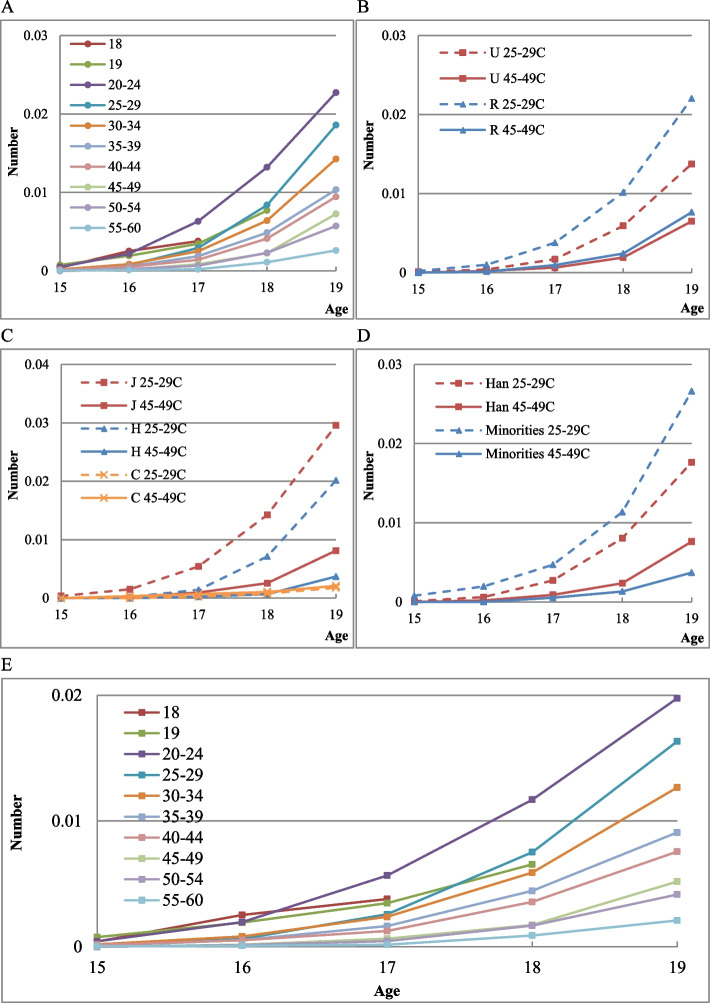
Age-specific cumulative number of induced abortions in a cohort. (5A) By cohort. (5B) By residence. (5C) By education. (5D) By ethnic group. (5E) Age-specific cumulative number of premarital induced abortion. Note: U and R represent Urban and Rural respectively; J, H, and C respectively represent junior middle school and below, high school education, college and above. Minority includes all other ethnic groups excluding Han Chinese

Figure 5 presents the cumulative number of adolescent induced abortions per woman by corresponding age in different cohorts. There is an upward trend for the cumulative number of adolescent induced abortions among Chinese women, and the number is higher for rural, less educated, and ethnic minority women.

For adolescents, induced abortions at the age of 15–19 are mostly premarital. Over 70% of all adolescent induced abortions are premarital in the current study. The cumulative number of premarital induced abortions also shows an increasing trend, as shown in Panel 5E. Women in the later birth cohort have more adolescent premarital induced abortions than that in the early birth cohort.

## Discussion

Based on data from a nationally representative survey, we examined the trend and characteristics of induced abortions among Chinese adolescent girls from period and cohort perspectives. Between 1996 and 2016, the proportion and rate of adolescent induced abortions first increased from 1996 to 2006 and then decreased. The mean age at which adolescent girls experienced induced abortions declined, which should be of significant concern.

Chinese children now enter puberty early and unmarried adolescents have engaged in more sexual activity over the past decades [19–21]. About 6.9% (10.8% in urban areas; 4.5% in rural areas) of young women aged 15–19 are sexually active [15, 22]. With the ideology of sex and marriage becoming more diversified, premarital sex has become more widely acceptable and prevalent among young people [23]. However, China’s family planning program has mainly focused on married couples in the last decades, giving young people limited access to related reproductive and contraception knowledge and services [23, 24].

The vast majority of adolescent induced abortions are due to unintended pregnancies, and the induced abortion proportion is much higher for unintended- than planned pregnancies. The proportion of women who had experienced adolescent unintended pregnancies is increasing in later birth cohorts, and it is higher for rural, less educated, and ethnic minority women. Meanwhile, the differences across groups increasingly diverge in the late birth cohort. Unintended pregnancies among adolescents are on the rise, with contraceptive failure or non-use being the leading cause [23]. The younger the age of the first intercourse, the higher the rate of non-use of contraceptive methods [25].

Adolescent girls have an unmet need for modern contraception and are at risk of unintended pregnancy [10, 26]. When urban adolescents unintendedly become pregnant, they are more likely to choose abortion. Compared with their urban counterparts, rural adolescents are more likely to experience unplanned pregnancies. This is mainly due to the fact that early marriage and childbearing still exist in rural areas. Although child marriage has been legally prohibited in China, it continues throughout the mainland, especially in rural areas [27, 28]. Marriage (usually informal unions) before 20 is more acceptable in rural areas than in urban areas, reducing the likelihood of induced abortion when rural adolescent girls unintendedly become pregnant.

The cumulative proportion of women who have experienced induced abortion at the age of 15–19 in a cohort is less than 2.5% but shows an upward trend. This proportion is higher for rural and less educated women. In the early birth cohort, the proportion of women with induced abortion experiences is higher among Han women, but it is higher among ethnic minority women in the late birth cohort. Furthermore, the difference becomes larger in the late birth cohort. The proportion of repeat induced abortions is also on the rise, with it being higher for rural, less educated, and ethnic minority women. This proportion markedly increased in the late birth cohort for women with a high school education or below. As mentioned above, urban women were more likely to have an abortion after an unintended pregnancy; however, the cumulative proportion of rural women who experienced an abortion was higher than urban women in a cohort.

China has been divided into a dual-structure society for decades, and there are obvious reproductive health resource and service disparities between the rural and urban areas [29]. Those living in rural areas typically have a lower quality of education and receive fewer reproductive health services [25, 30, 31]. Compared with their rural counterparts who have limited education, women with higher education have stayed in school for a longer period and have fewer opportunities to make contact with society and engage in sexual intercourse [23, 32].

The cumulative number of induced abortions in a cohort also increased with later cohorts. In the late birth cohort, the number is higher for women who live in rural areas, are less educated, and come from ethnic minorities. The majority of induced abortions are premarital. In traditional Chinese society, open discussions about sex in mainland China is largely considered taboo [33]. Influenced by traditional Confucianism, Chinese society valued girls’ virginity and denounced premarital pregnancies ever since the ancient times [34, 35]. Although premarital sex has become increasingly acceptable in China, adolescent pregnancy still carries a social stigma in current Chinese society. Young women reported that they would not want their parents to know about their premarital sexual behavior and disgrace the family, leading to a tendency for some adolescent girls to delay seeking help from adults or turn to private hospitals for abortion services [3, 36]. The upward trend in adolescent induced abortion and the decline in the age at which induced abortions occur should be a cause for significant concern.

A document newly issued in 2022 by China Family Planning Association [37] pointed out that the reproductive health service should be further promoted to focus on the prominent reproductive health problems among specific groups, including adolescents. Moreoever, induced abortion interventions should be conducted among single women. All sectors of society were enjoined to pay attention to adolescent reproductive health issues, promote sexual education and reproductive health services, and reduce unintended pregnancies and repeat induced abortions among adolescents.

Firstly, it is urgent to promote sexual education among adolescents. There is strong inernational evidence that comprehensive sex education programs lead to safer sexual behaviors [38, 39], and that sex education can reduce unwanted pregnancies and associated abortions, as well as protect young people from sexually-transmitted infections [18, 40]. Schools should provide sexual and reproductive health knowledge, while parents, society, and related medical institutions should take it as an obligation to educate adolescents on reproductive health and contraceptive knowledge, as well as raise their awareness on contraception. China needs to develop and widely disseminate online sex education with practical, age-appropriate content [39]. Schools and kindergartens should also provide age-appropriate sex education for minors.

Secondly, accessible reproductive services should be strengthened. Attention should be paid to the reproductive health and contraceptive needs of adolescents under the age of 20, especially those at high risk of unintended pregnancies and induced abortion. These can include suitable, affordable, and accessible services involving regular consultations for reproductive health and contraception. Moreover, measures should be taken to narrow the gap between urban and rural adolescents, and Han and ethnic-minority adolescents.

There are still several limitations to this study. First, the induced abortions that were experienced at the age of 15–19 may be underreported due to recall bias, especially among women in early birth cohorts [35, 41]. In addition, there exists underreporting due to the sensitive nature of questions on adolescent induced abortions. Young women may feel ashamed to report their experiences of pregnancy and abortion during adolescence. Second, education level was only measured at the time of the survey instead of with a time-varying variable. We assume that education levels are unlikely to change significantly due to most people finishing their school before marriage and childbearing [17]. However, some young women are also likely to pursue further education after having an induced abortion before the age of 20. Therefore, there may be a measurement error in education level at the time of induced abortion. Finally, the results and conclusions in this study may not be generalizable to other cultural settings since the data were based on the Chinese context.

## Conclusion

The prevalence of women who have experienced induced abortion at the age of 15–19 is increasing and the age at which women experienced induced abortion is declining, which should arouse concern. There are significant differences in induced abortions among adolescent girls with different characteristics. Adolescent girls who are in later birth cohort, who live in rural areas, who are less educated, or are from ethnic minorities, are more likely to undergo induced abortions. More appropriate educational efforts and interventions are in dire need to reduce the incidence of adolescent induced abortions.

### Supplementary Information

**Additional file 1.**

## Data Availability

All tabulated data relevant to this study are included in the article.

## Footnotes

1 According to the “Regulations on Statistical Classification of Urban and Rural Areas” issued by the National Bureau of Statistics, the Chinese government divided the area into urban and rural areas based on the public facilities, residential facilities, and other facilities that have been built or are under construction.
