# Supplementary material for: Induced abortions among Chinese adolescent girls
Source: BMC Womens Health. 2023 Nov 13;23:597. doi: 10.1186/s12905-023-02754-w (PMC10644521; doi:10.1186/s12905-023-02754-w)
Supplement: Supplementary file 1 — Additional file 1. [file 12905_2023_2754_MOESM1_ESM.docx]

**Supplemental Table 1. Overall trend of induced abortions among women aged 15-19.**

| Year | Proportion  (%) | Rate  (‰) | Mean age of induced abortions among first pregnancies | Mean age of induced abortions |
| --- | --- | --- | --- | --- |
| 1996 | 12.02 | 2.06 | 18.79 | 18.77 |
| 1997 | 11.79 | 1.90 | 18.80 | 18.92 |
| 1998 | 15.01 | 2.30 | 18.80 | 18.90 |
| 1999 | 13.08 | 2.02 | 18.66 | 18.70 |
| 2000 | 14.46 | 2.30 | 18.51 | 18.67 |
| 2001 | 11.44 | 1.96 | 18.51 | 18.66 |
| 2002 | 14.14 | 2.18 | 18.51 | 18.58 |
| 2003 | 18.75 | 2.86 | 18.81 | 18.88 |
| 2004 | 17.77 | 2.93 | 18.73 | 18.77 |
| 2005 | 16.84 | 3.03 | 18.73 | 18.82 |
| 2006 | 18.46 | 4.08 | 18.84 | 18.86 |
| 2007 | 15.34 | 3.50 | 18.83 | 18.87 |
| 2008 | 15.71 | 4.59 | 18.76 | 18.82 |
| 2009 | 15.46 | 5.30 | 18.63 | 18.81 |
| 2010 | 16.86 | 6.66 | 18.56 | 18.75 |
| 2011 | 15.56 | 5.80 | 18.58 | 18.74 |
| 2012 | 16.26 | 6.40 | 18.26 | 18.51 |
| 2013 | 14.63 | 5.20 | 18.34 | 18.59 |
| 2014 | 11.55 | 3.96 | 18.51 | 18.68 |
| 2015 | 14.72 | 3.84 | 18.48 | 18.68 |
| 2016 | 11.15 | 2.76 | 18.32 | 18.64 |
